# The optimal timing of FDG-PET/CT in non-small cell lung cancer diagnosis and staging in an Australian centre

**DOI:** 10.1186/s12890-021-01564-w

**Published:** 2021-07-01

**Authors:** Anne Johnson, Richard Norman, Francesco Piccolo, David Manners

**Affiliations:** 1 St John of God Midland Public and Private Hospitals, Midland, WA Australia; 2 School of Population Health, Curtin University, Bentley, WA Australia

## Abstract

**Background:**

Clinical practice guidelines and re-imbursement schedules vary in the recommended timing of FDG-PET/CT in the diagnostic evaluation of suspected or confirmed lung cancer. The aim was to estimate the probability of requiring more than one invasive test to complete diagnosis and staging in non-small cell lung cancer if FDG-PET/CT was used prior to initial biopsy (FDG-PET/CT First) compared to current Australian funding criteria (CT First).

**Methods:**

Single-centre retrospective study of individuals with pathologically confirmed NSCLC without evidence of metastatic disease on baseline computed tomography (CT) of the chest. Decision tree analysis based on diagnosis and staging approaches estimated the probability of requiring more than one invasive biopsy. A Monte Carlo analysis with 1000 simulations was used to estimate decision tree precision.

**Results:**

After exclusions, 115 patients were included with median (IQR) age of 71 (63–79) and 55.6% were male. The majority of cases were early stage (Stage I 43.5%, Stage II 19.1%) and adenocarcinoma (65.2%) histological subtype. The estimated probability of requiring more than one invasive biopsy with FDG-PET/CT prior was 0.12 compared to 0.19 when using the base case CT First scenario. Using the Monte Carlo analysis, the mean (95% CI) probability using the FDG-PET First approach was 0.15 (95%CI 0.12–0.20) versus 0.20 (95% CI 0.15–0.27) for the CT First approach. Only 7.8% had CT Chest-occult metastatic disease on FDG-PET that was accessible by percutaneous biopsy.

**Conclusion:**

FDG-PET/CT performed prior to initial biopsy may reduce the proportion of people with NSCLC who require more than one biopsy attempt, but the clinical significance and overall cost-utility requires evaluation.

**Supplementary Information:**

The online version contains supplementary material available at 10.1186/s12890-021-01564-w.

## Background

Lung cancer is the leading cause of cancer-related mortality worldwide [1]. With small pulmonary nodules being increasingly encountered in clinical practice and lung cancer screening cohorts, strong diagnostic guidelines are vital for accurate and efficient diagnosis to facilitate early treatment and reduce unnecessary investigations. Computed Tomography (CT) is an excellent tool for detection and localization of pulmonary nodules; however, it has a poor specificity for further characterization [2]. 18-fluorine-Fluorodeoxyglucose-positron emission tomography/computed tomography (FDG-PET/CT) is a powerful imaging modality for assessing suspected and confirmed malignancy [3]. One meta-analysis determined that for predicting lung malignancy, FDG-PET/CT has an estimated sensitivity and specificity of 96.8% and 77.8%, respectively [4]. A subsequent study by Budak et al*.* [5] demonstrated sensitivity of 94% of FDG-PET/CT in detecting malignancy, and additionally demonstrated a change in treatment plan in 34% of patients based on FDG-PET/CT. Furthermore, the development of a prediction tool for assessment of adenopathy in lung cancer (HAL score) highlighted that FDG-PET/CT improved the accuracy estimate significantly, with the incremental value of information greater in those with N0 disease by CT, highlighting the importance of discordant CT and FDG-PET/CT results [6].

These benefits of FDG-PET/CT in the evaluation of suspicious pulmonary nodules are reflected in international guidelines published by the National Comprehensive Cancer Network and the British Thoracic Society, which recommend FDG-PET/CT prior to further diagnostic intervention as detection of metastases on FDG-PET/CT can direct an alternative route to obtaining tissue for pathological diagnosis [7–9]. Australian clinical guidelines suggest FDG-PET/CT can be used prior to biopsy in order to guide biopsy as well as to stage disease [10]. However, current Australian funding provides subsidies only for (i) evaluation of a solitary pulmonary nodule where the lesion in unsuitable for biopsy or pathological characterisation has failed or (ii) staging of Non-Small Cell Lung Cancer (NSCLC) with proven tissue diagnosis, where curative treatment is planned [11].

There is a lack of literature addressing the optimal timing of FDG-PET/CT in the lung cancer diagnostic imaging pathway in Australian centres. Further evaluation into its role prior to biopsy in an Australian setting may be useful to advise future investigation pathways for suspected lung cancer and government scheduling criteria. The aim of this study was, in those with NSCLC, to compare the probability of requiring more than one invasive biopsy to complete diagnosis and staging when FDG-PET/CT is used before or after histological confirmation of NSCLC.

## Methods

This single-centre retrospective study analysed data from a pre-existing database of patients at St John of God Midland Public/Private Hospital between 13th April 2016 and 31st December 2019 who had undergone FDG-PET/CT scanning for investigation of lung cancer or lung nodules. Patients aged over 18 were included if they had a confirmed tissue diagnosis of NSCLC and both baseline CT and either baseline or pre-treatment staging FDG-PET/CT. Patients were excluded if no baseline CT Chest or FDG-PET/CT was available, baseline CT findings were consistent with radiological stage IV disease, no tissue diagnosis of NSCLC or age < 18 years old.

For each patient meeting the inclusion criteria, two reviewers independently assessed each CT to confirm the presence of a solid/semi-solid lung nodule without radiological evidence of stage IV disease. The reviewers assessed centrality of the tumour, suitability for radial endobronchial ultrasound (EBUS) defined by lesion > 2 cm in size and presence of air-bronchogram to lesion, presence of hilar and/or mediastinal lymphadenopathy and presence of accessible pathological supraclavicular lymphadenopathy. Then each FDG-PET/CT was assessed for hilar or mediastinal lymph node abnormalities or the presence of likely distant metastases as alternative site to biopsy. Distant metastastic sites deemed accessible with acceptable risk for the purpose of this study included supraclavicular or axillae lymph nodes, liver, bone (only if bony cortex was disrupted) and adrenals. Using this data, the three HAL scores (Full HAL model and HAL models including age and CT findings only [HAL eTable 8] and age, CT and FDG-PET/CT findings [HAL eTable 6] were calculated for each patient [6]. The HAL score is a multivariable logistic regression model developed and validated from participants the U.S. based Aquire bronchoscopy registry that uses age, mediasitanal and hilar lymphadenopathy on CT chest and FDG-PTE/CT and histological NSCLC subtype to estimate the probability of detecting N2 or N3 metastatic spread via EBUS. Where interpretation of imaging differed between the two reviewers, a third clinician reviewed the imaging for consensus opinion.

## Statistical analysis and decision tree analysis

Inter-observer agreement was calculated with kappa for dichotomous outcomes and Pearson’s R for continuous variables. McNemar’s test for paired samples was used to compare dichotomous outcomes. Confidence intervals for proportions were estimated using the continuity adjustment. Analysis was performed on SAS OnDemand for Academics (SAS Institute, Cary, U.S.)

To model the flow of patients through the investigation process, a decision tree was constructed in TreeAge Pro (TreeAge Software, Williamstown, U.S.) based on a pre-determined diagnostic evaluation approach developed by the study authors. This approach models the patient pathway as a comparison between multiple options, each of which leads to a series of chance nodes. The final tree is presented in Additional file 1: Appendix A. Probabilities were then assigned to each of the chance nodes using either our own primary data or obtained from published literature identified by a non-systematic literature review. The data sources for each chance node are presented in Table 1. Each parameter used in the decision tree was subject to uncertainty. Therefore, we conducted a probabilistic sensitivity analysis. In this, we assigned a distribution to each of the parameters. These were typically triangular reflecting that we had a point estimate for each probability, and a higher and lower threshold for what was plausible. We then conducted a Monte Carlo analysis with 1000 simulations. This draws from each distribution independently and estimates the proportion of patients requiring further investigation in each simulation. Using the distribution of the 1000 simulations, we constructed a 95% confidence interval around the proportion of patients requiring more than one invasive biopsy using either diagnostic approach.

**Table 1:** Decision tree chance nodes with estimates

Variable	Estimate (%)	Plausible range/95% confidence interval	Data source
CT first decision tree			
Supraclavicular lymphadenopathy	1.7%	0.3–6.7%	Study cohort
USS-guided lymph node biopsy sensitivity	93%	90–96%	Extrapolated from Han et al*.* [12]
HAL model eTable 8 > 10%	51%	42–60%	Study cohort
Nodule accessible by radial EBUS	57%	44–69%	Study cohort
Radial and Linear EBUS sensitivity	73%	70–76%	Published data [13]
Linear EBUS sensitivity	89%	46–97%	Published data [14, 15]
Ct-guided lung biopsy sensitivity	93%	90–97%	Published data [12]
Distant metastatic disease on FDG PET	38%	27–51%	Study cohort
Full HAL model > 10	21%	10–37%	Study cohort
Accessible met disease OR FDG PET positive hilar LN OR Nodule accessible with radial EBUS	51%	38–64%	Study cohort
PET first decision tree			
Accessible metastatic disease	7.9%	4.2–14%	Study cohort
Percutaneous image-guided biopsy sensitivity	93%	90–96%	Published data [16]
FDG PET positive hilar LN OR HAL > 10%	42%	33–52%	Study cohort
Nodule accessible by radial EBUS	64%	50–77%	Study cohort
Radial and linear EBUS sensitivity	73%	70–76%	Published data [13]
Linear EBUS sensitivity	89%	46–97%	Published data [14, 15]
CT-guided lung biopsy sensitivity	93%	90–97%	Published data [12]
Full HAL model > 10%	3.3%	0.9–11%	Study cohort
Nodule accessible by radial EBUS	44%	33–57%	Study cohort

*CT* Computed tomography, *EBUS* Endobronchial ultrasound, *HAL* Help with Assessment of Lymphadenopathy Model^6^. *USS* Ultrasound

## Results

**Fig. 1:**
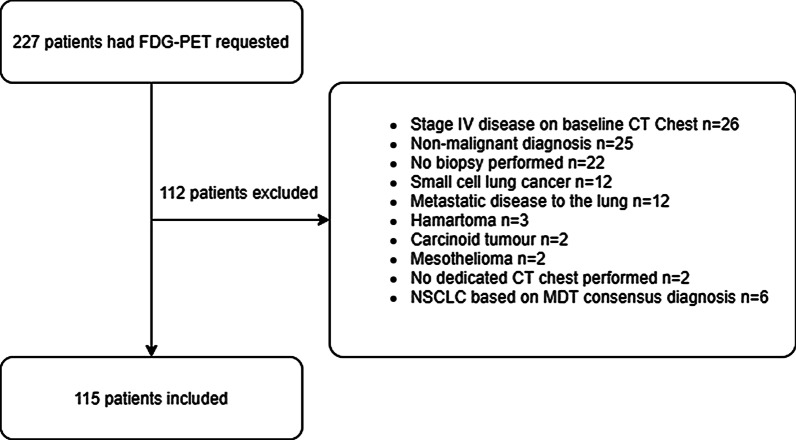
Reasons for patient exclusions

FDG-PET/CT scans were performed on 227 patients of whom 112 we excluded, primarily due to having stage IV disease on baseline CT chest, non-malignant diagnoses or no histological confirmation of NSCLC, leaving 115 patients included (Fig. 1). Baseline demographics including participant age, gender, lung cancer staging and histological subtype are presented in Table 2. Inter-observer agreement (Table 3) on decision tree points ranged from minimal for whether the primary lesion was accessible by radial EBUS (kappa 0.291, 95%CI 0.163–0.420) to strong for the HAL model calculation (Pearson’s R 0.872, 95% CI 0.821–0.910).

**Table 2:** Baseline participant characteristics

Demographic	% (n) or Median(IQR)
Age (years)	71 (63–79)
Gender	
Male	55.6% (64)
Female	44.4% (51)
Final NSCLC clinical stage	
I	43.5% (50)
II	19.1% (22)
IIIa	14.8% (17)
IIIb	10.4% (12)
IV	12.2% (14)
NSCLC histology	
Adenocarcinoma	65.2% (75)
Squamous cell carcinoma	28.7% (33)
Non-small cell other	6.1% (7)

*NSCLC* Non-small cell lung cancer

**Table 3:** Inter-observer agreement for decision tree chance nodes

Decision tree chance node measurement	Kappa	95% CI
Supraclavicular lymphadenopathy	1.00	1.00–1.00
Radial EBUS accessible	0.291	0.163–0.420
Distant metastatic disease on FDG-PET/CT	0.661	0.428–0.895
Accessible metastatic disease	0.696	0.415–0.977
Hilar FDG-PET positive lymph nodes	0.626	0.469–0.783
HAL full model	0.873	0.821–0.910

**Fig. 2:**
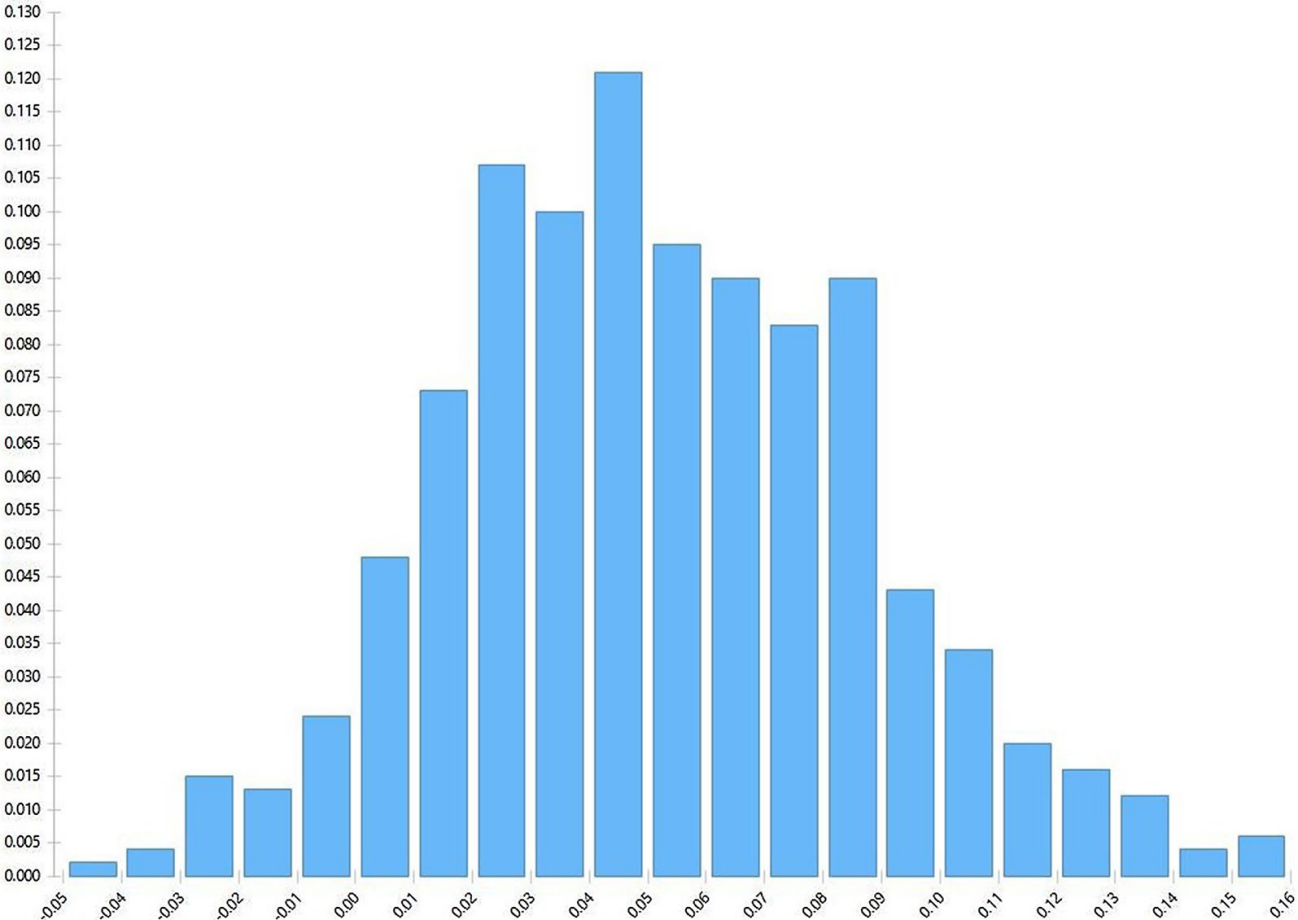
Monte Carlo Probability Distribution Histogram. This histogram shows the distribution of the difference in estimated probabilities of requiring more than one invasive test (“CT-First” minus “FDG-PET/CT First”) with 1000 repetitions of the decision tree using a Monte Carlo analysis

The estimated probability of requiring more than one invasive biopsy attempt using base-case test sensitivity assumptions was 0.192 when using the “CT First” diagnostic approach and 0.121 when using the “FDG-PET/CT First” approach. Therefore, we estimate that FDG-PET/CT prior to initial biopsy would reduce the risk of requiring more than one invasive biopsy by 7.1%. Using the Monte Carlo analysis, the mean probability of requiring more than one invasive biopsy was 0.20 (95% CI 0.15–0.27) for the “CT First” approach and 0.15 (95%CI 0.12–0.20) when using the “FDG-PET/CT First” approach. The Monte Carlo probability distribution histogram (Fig. 2) shows that the “FDG-PET/CT First” approach had a lower probability of requiring more biopsies in 93.4% of simulations. The mean (± SD) difference in estimated probabilities was 0.05 (± 0.035).

Fourteen (12.2%) participants had CT Chest–occult metastatic disease identified on FDG-PET scan, yet only nine (7.8%) of these had accessible disease by a percutaneous approach including four individuals with adrenal metastases, two accessible lymph node metastases and isolated individuals with destructive osseous, liver or duodenal metastases. The remainder had either osseous metastases with an intact bony cortex (four individuals) or cerebral metastases (one individual). In those without supraclavicular lymphadenopathy or accessible metastatic disease on FDG-PET, there was a significant decrease in the proportion of patients who may require linear EBUS for mediastinal staging (HAL score > 10%) when the HAL e6 model (including FDG-PET) was used compared to HAL e8 model (age and CT-Chest findings only) [35.6% vs 45.2%, *p* = 0.045].

The results of the sensitivity analysis of chance nodes of the decision tree are presented in Table 4. Increasing sensitivity of radial EBUS and reducing sensitivity of linear EBUS increased the difference in probability of requiring more than one test using the different approaches while increasing the HAL threshold for mediastinal staging decreased the difference in probability.

**Table 4:** Decision tree chance node sensitivity analysis

Chance node	CT-guided Lung Biopsy Sensitivity	Radial EBUS sensitivity	Linear EBUS sensitivity	Radiologically guided percutaneous biopsy sensitivity	HAL threshold for mediasitinal staging	“CT-First” Probability of requiring more than one diagnostic test	“FDG-PET/CT First” Probability of requiring more than one diagnostic test	Difference in probabilities	% Change from base case
*Base case*									
	93%	73%	89%	93%	10%	0.192	0.121	0.071	N/A
*CT-guided Lung Biopsy Sensitivity*									
	90%					0.205	0.131	0.074	3.4%
	96%					0.180	0.104	0.076	7.2%
*Radial EBUS Sensitivity*									
		69%				0.204	0.129	0.075	5.3%
		80%				0.229	0.101	0.128	79.8%
*Linear EBUS Sensitivity*									
			Mean HAL score (25.8%)			0.330	0.209	0.121	69.6%
			46%			0.286	0.181	0.105	47.3%
			97%			0.175	0.110	0.065	− 8.8%
I*mage-Guided Percutaneous Biopsy Sensitivity*									
				90%		0.193	0.123	0.070	− 2.6%
				96%		0.192	0.119	0.073	2.6%
*HAL threshold for systematic pre-treatment mediastinal staging*									
					17.5%	0.140	0.101	0.039	− 45.5%
					25%	0.128	0.101	0.027	− 62.0%

## Discussion

Our retrospective decision-tree analysis suggests that, in those with NSCLC and without evidence of stage IV disease on baseline CT chest, a diagnostic and staging approach starting with whole body FDG-PET/CT is less likely to require more than one biopsy attempt than an approach that utilises FDG-PET/CT after pathological confirmation or failed biopsy attempt. This results supports Australian and international guidelines that suggest FDG-PET/CT prior to initial biopsy [9, 10]. Extrapolating the result implies that approximately one out of 14 people with NSCLC would avoid additional biopsy if FDG-PET/CT was used prior to initial biopsy. The clinical significance of this result is uncertain. The potential benefit comes at the expense of additional FDG-PET/CT scans performed on individuals with baseline imaging of suspected lung cancer who subsequently are confirmed to have benign or non-NSCLC malignant diagnoses. In our cohort, 28% of patients fit into this category, who may have undergone low-value FDG-PET/CT scans.

FDG-PET/CT is more accurate than other modalities for the detection of metastatic disease with pooled sensitivity and specificity of 0.77 (95% CI: 0.47–0.93) and 0.95 (95% CI: 0.92–0.97) respectively [17]. The rate of CT-Chest occult metastatic disease identified on FDG-PET/CT in our cohort was 12.2%, lower than previously described in a similar Australian cohort who were undergoing radiotherapy (19%, *p* = 0.002), yet similar to other prospective studies [18–20]. While this is clinically important in the pre-treatment assessment of NSCLC for accurate clinical staging, the hypothesised benefit of FDG-PET/CT in pre-diagnosis assessment is to guide biopsy of the most distant site of suspected disease—an approach that simultaneously confirms diagnosis and staging. Approximately one third (5/14) of CT Chest-occult metastatic lesions were not amenable to percutaneous biopsy, reducing the value pre-diagnosis FDG-PET/CT to guide biopsy approach.

Herder et al*.* performed a prospective randomised-control trial in individuals with CXR abnormalities suggestive of lung cancer in which a diagnosis and staging approach using initial FDG-PET without CT was compared to traditional work-up (TWU). There was no difference in the primary outcome of total number of tests and procedures to finalize staging and define operability (mean 7.90 for initial FDG-PET vs 7.88 for TWU). There was also no difference the number of invasive tests (mean 0.85 vs 0.96, *p* = 0.18), but there were fewer tests requiring general anaesthesia (mean 0.59 vs 0.78, *p* = 0.0074). Costs of staging investigations were similar in both study arms. Factors that may reduce the generalizability to current practice include FDG-PET scans being performed without concurrent CT scanning, inclusion criteria being based on CXR abnormalities suggestive of lung cancer and the widespread introduction of endobronchial ultrasound technology to perform mediastinal sampling.

Other prospective randomised-control trials have investigated the role of FDG-PET in avoiding futile thoracotomy, defined as a composite endpoint of thoracotomy for benign disease, pathologicaly proven mediastinal lymph node involvement, explorative thoracotomy or recurrent disease or death within one year. The PLUS study suggested standalone FDG-PET reduced futile thoracotomy from 41 to 21% (*p* = 0.003) translating to one patient avoiding futile thoracotomy for every five patient undergoing FDG-PET (95%CI 3–14) [19]. Costs were reduced given fewer thoracotomies and associated hospital bed-days [21]. This result was repeated by Fisher et al*.* who reported that FDG-PET/CT reduced futile thoracotomy from 52 to 35% (*p* = 0.05) [22]. The number of individual invasive investigations prior to treatment were similar between the two groups.

Mac Manus et al*.* performed a similar study where 167 patient with confirmed NSCLC with Stage I-III disease based on CT were allocated clinical staging with or without FDG-PET/CT [18]. The proportion of CT-occult distant metastasis increased with FDG-PET/CT staging, but the impact on biopsy approach was not discussed. The ongoing “PET/CT FIRST” study is using a similar matched study design where the recommended biopsy test will be based on the pre- and post-FDG-PET/CT clinical information [23].

The aims and contextual rationale for our study is framed by current regulatory funding in Australia for FDG-PET/CT scans, which may not apply in other jurisdictions. In Australia, the Medicare Benefits Schedule (MBS) provides criteria for government subsidy of medical investigations and non-pharmaceutical treatment. The current MBS criteria for FDG-PET/CT in lung cancer are for (i) evaluation of a solitary pulmonary nodule where the lesion is considered unsuitable for transthoracic fine needle aspiration biopsy, or for which an attempt at pathological characterisation has failed or (ii) the staging of proven NSCLC, where curative surgery or radiotherapy is planned [11]. FDG-PET/CT scanning performed to guide initial biopsy in suspected lung cancer is not specifically subsidised. More robust, prospective evidence, accompanied by cost-utility analysis will be required to update MBS FDG-PET/CT scan criteria to include pre-biopsy in those with suspected lung cancer, but the feasibility of such a study is unclear. A sample size calculation using data from the current study for the primary outcome of the proportion who people needing more than one invasive investigation would require a minimum of 836 participants (19% vs 12%, power 0.80, alpha 0.05). There may be additional benefits of pre-biopsy FDG-PET/CT, such as reduced time to treatment, but faster treatment may not necessarily be associated with improved outcomes and this needs to be balanced by the additional cost and radiation exposure of FDG-PET/CT on individuals who do not have NSCLC [24]. Clinicians who practice outside of current Australian funding requirements may use these results to weigh potential benefits of FDG-PET/CT prior to initial biopsy, such as fewer repeat biopsies on individuals with NSCLC with potential downsides including cost and radiation exposure of FDG-PET/CT scans on individuals without malignancy.

Many limitations of the current study need acknowledging. These include the retrospective nature and lack of a pre-determined sample size. Most individuals (62.6%) had early stage (I or II) NSCLC, which may have reduced the probability of FDG-PET/CT detected accessible metastatic disease. An alternative inclusion criteria such as baseline CT-Chest evidence of stage II or III disease may lead to an enriched population where FDG-PET/CT prior to biopsy may have more benefit at reducing repeated biopsy attempts. We assumed that all participants would have been suitable for all recommended invasive investigations, yet our known real-world experience is that individual patients may have varied diagnostic pathways due to technical and patient-related factors that were not accounted for in our study. For example, all pulmonary nodules/masses were deemed suitable for CT-guided transthoracic biopsy. Additionally, our clinical decision tree used the HAL model to guide need for systematic pre-operative mediastinal. The 10% probability for detecting N2/3 lymph node involvement with EBUS threshold suggested by the HAL model authors has (i) not been prospectively validated, (ii) compared to existing recommended criteria for systematic mediastinal staging or (iii) incorporated into clinical practice guidelines. The choice of primary outcome was a pragmatic decision that balanced feasibility of statistical analysis using a simple decision tree with clinical relevance, but the real-world importance of patients needing more than one invasive biopsy requires further consideration.

## Conclusion

FDG-PET/CT scans performed prior to initial biopsy attempts may reduce the proportion of people with NSCLC who require more than one biopsy attempt, but the clinical significance and overall cost-utility needs further prospective evaluation.

## Supplementary Information

**Additional file 1.** Decision tree layout for “FDG-PET/CT First” and “CT First” diagnostic approaches.

## Data Availability

The datasets used and/or analysed during the current study are available from the corresponding author on reasonable request.
