# Supplementary figures and images for: The optimal timing of FDG-PET/CT in non-small cell lung cancer diagnosis and staging in an Australian centre
Source: BMC Pulm Med. 2021 Jul 1;21:209. doi: 10.1186/s12890-021-01564-w (PMC8252249; doi:10.1186/s12890-021-01564-w)

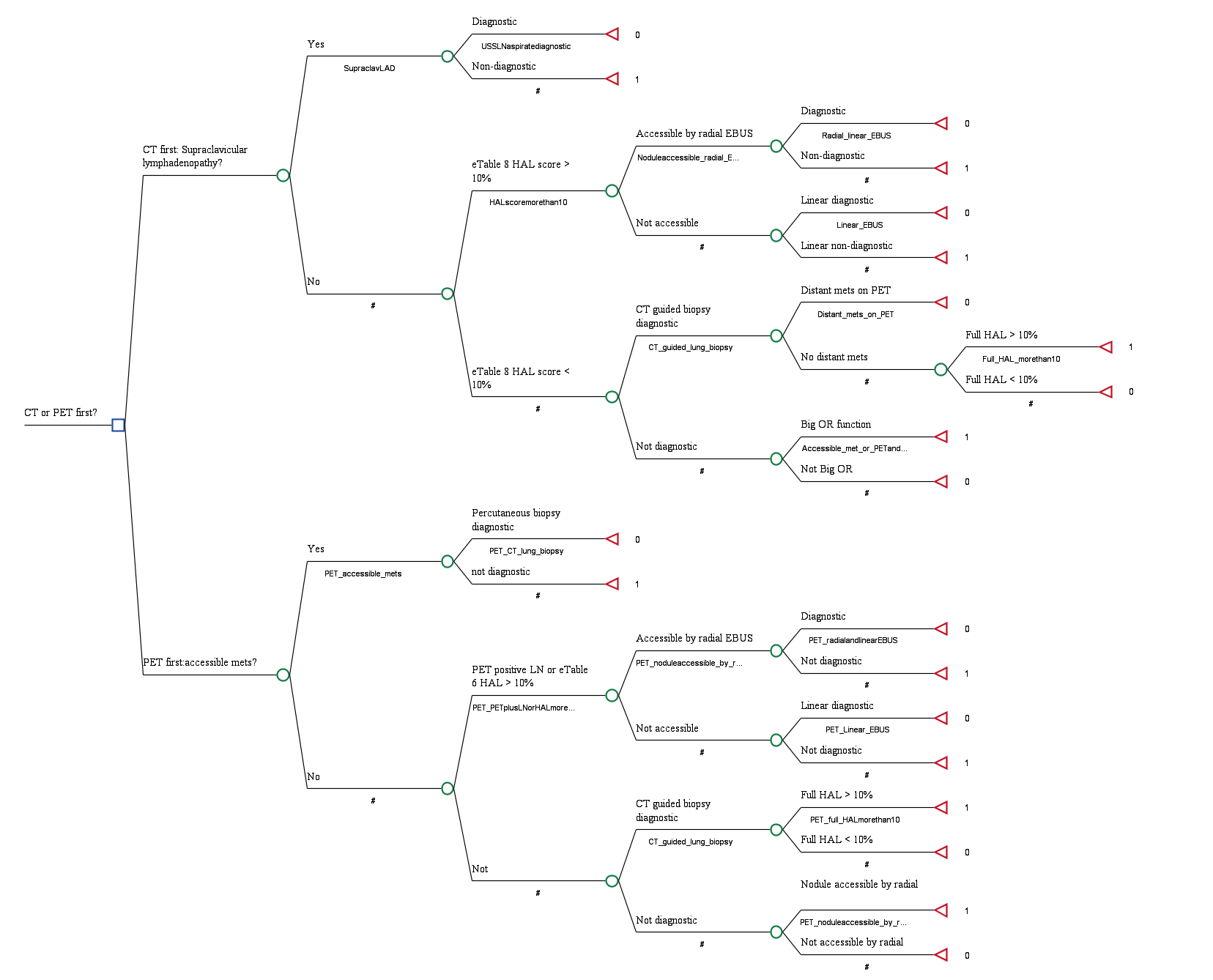

Supplement: Supplementary file 1 — Additional file 1. Decision tree layout for “FDG-PET/CT First” and “CT First” diagnostic approaches. [file 12890_2021_1564_MOESM1_ESM.png]
